# Spreading Behavior of Non-Spherical Particles with Reconstructed Shapes Using Discrete Element Method in Additive Manufacturing

**DOI:** 10.3390/polym16091179

**Published:** 2024-04-23

**Authors:** Tengfang Zhang, Dan Chen, Hui Yang, Wei Zhao, Yunming Wang, Huamin Zhou

**Affiliations:** 1 State Key Laboratory of Materials Processing and Die & Mould Technology, School of Materials Science and Engineering, Huazhong University of Science and Technology, Wuhan 430074, China; zhangtengfang2009@126.com; 2 College of Advanced Interdisciplinary Studies & Hunan Provincial Key Laboratory of Novel NanoOptoelectronic Information Materials and Devices, National University of Defense Technology, Changsha 410073, China; 18874050884@163.com; 3 Nanhu Laser Laboratory, National University of Defense Technology, Changsha 410073, China; 4 Wuhan Second Ship Design and Research Institute, Wuhan 430000, China; smilebuxi@163.com (H.Y.); xyzwei159@126.com (W.Z.)

**Keywords:** additive manufacturing, spreading behaviors, non-spherical particles, reconstruction shape

## Abstract

The spreading behavior of particles has a significant impact on the processing quality of additive manufacturing. Compared with spherical metal material, polymer particles are usually non-spherical in shape. However, the effects of particle shape and underlying mechanisms remain unclear. Here, the spreading process of particles with reconstructed shapes (non-spherical particles decomposed into several spherical shapes by stereo-lithography models) are simulated by integrating spherical particles with the discrete element method. The results show that more cavities form in the spreading beds of particles with reconstructed shapes than those of spheres with blade spreading. Correspondingly, particles with reconstructed shapes have lower packing densities, leading to more uniform packing patterns. Slow propagation speeds of velocity and angular velocity lead to “right-upwards” turning boundaries for particles with reconstructed shapes and “right-downwards” turning boundaries for spherical particles. Moreover, as the blade velocity increases, the packing density decreases. Our calculation results verify each other and are in good agreement with the experiment, providing more details of the behavior of non-spherical particles before additive manufacturing. The comprehensive comparison between polymer non-spherical particles and spherical particles helps develop a reasonable map for the appropriate choice of operating parameters in real processes.

## 1. Introduction

Powder bed-based additive manufacturing (AM) is widely adopted due to its ability to fabricate complex and high-quality components [1,2]. During the process, powder is spread onto the working surface by a blade or roller spreader to form a thin powder layer with a thickness of several particle sizes, which is then irradiated by a laser or electron beam to melt or sinter the selected area [3]. In this aspect, the spreading quality of a thin powder layer plays a crucial role in the subsequent melting process [4]. Achieving a uniform, dense powder layer is beneficial to minimize spreading defects (such as spatter, keyholes, and denudation), thus enhancing mechanical properties and surface quality [5,6].

Thus, to control and optimize the quality of spreading beds, the characterization of powder behaviors should be the clarified. Researchers applied both experimental and simulation methods to investigate the flowability of powder used in AM, in which different operational factors were examined [7]. Meier et al. studied the angle of repose of Ti-6Al-4V powder considering the effect of adhesion force defined by the surface energy between fine powder particles [8]. Lee et al. explored the effect of mass scaling for small time steps of nano-sized IN718 powder particles [9]. Wang et al. investigated the effect of the blade geometry [10] and cohesive force [10] of the spreading process of metal particle. Nan et al. explained the effect of gap height and blade spreading speed [11] on the evolving shear band and mass flow rate of 316L powder particles and the formation frequency and size of empty patches [12,13]. Fouda et al. mentioned three mechanisms including shear-induced dilation caused by initial spreader movements, dilation, and rearrangement under the gap and the inertia of the particles by simulating the spreading of spherical, mono-sized, non-cohesive titanium alloy (Ti6AlV4) particles [14]. Snow et al. provided four metrics to describe the spreading quality, which were the percentage of the build plate covered by spread powder, the rate of powder deposition, the average avalanching angle of the powder, and rate of change in the avalanche angle by simulating Al-10Si-0.5Mg particles [15].

Through reviews of the literature, the effects of operation factors and mechanisms have been widely investigated for metal powder particles in previous years. Meanwhile, due to the nature of metal powder, sphere-shaped particles have been utilized in most simulations [16]. Recently, simulations involving polymer powder have emerged, which are not limited to the metal powder field, for the reason that the properties of polymer materials are far different from those of metal materials [17]. Harie et al. investigated the spreading process of different-shaped PMMA particles, illustrating that an increased aspect ratio would destroy the uniformity of the powder bed and decrease the bed density [18]. Chen et al. examined the effect of fiber particles and found that increasing the fiber length reduces the spreadability of mixed powders and decreases the packing density [19], and they proposed a deposition mechanism and percolation effect of bimodal powder particles [20]. Cheng et al. [21] and Si et al. [22] examined the effect of different operation factors and mechanisms of spherical polymers. However, the investigation of non-spherical polymer particles is rather rare compared to those of spherical particles. The spreading process and the underlying mechanisms influencing the processing properties are not deeply understood.

Herein, we present a numerical investigation of the spreading behavior of non-spherical PA6 particles with reconstructed shapes (non-spherical particles decomposed into several spherical shapes by stereo-lithography models) in AM using the discrete element method (DEM). This calculation can be generalized to simulate other polymer non-spherical particles according to their different physical properties. The difference in powder-spreading behavior between sphere particles and particles with reconstructed shapes is compared, including the packing pattern, density, and velocity distribution. Meanwhile, the effect of spreading speed, blade velocity, and surface energy is explored to gain further understanding of the particle spreading mechanism. The detailed relative mechanisms at the particle scale, including particle blocking, force arches, particle velocity distribution, and cohesion force, are discussed. The understanding of flow behavior regarding non-spherical particles can realize uniform powder layers and improve the mechanical performances of AM parts.

## 2. Simulation Method and Conditions

### 2.1. Governing Equation

**Figure 1:**
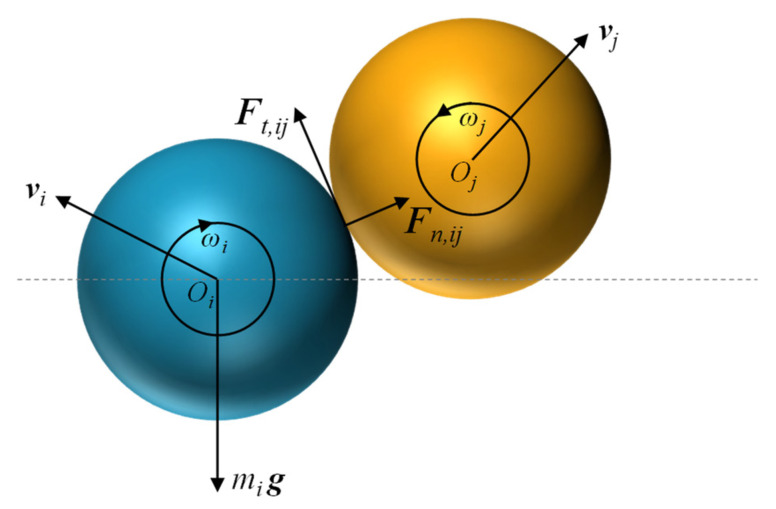
The force exerted on particle *i* in contact with particle *j* in DEM simulation.

The discrete element method is used in this work to capture the movement of particles with reconstructed shapes, which contains two typical motions: translational and rotational. During movement, the momentum is exchanged when particles have collisions with their neighboring particles or the hopper wall. The governing equation is listed here to explain the particle–particle interaction at the contact point. The translational and rotational motion of a given particle, *i*, with mass *m_i_* and moment of inertia *I_i_* are governed by Newton’s second law of motion in the DEM [23], written as follows in Equations (1) and (2) (Figure 1):

(1) $$m_{i}\frac{dv_{i}}{dt}=\sum_{j=1}^{k_{i}}\left(F_{n,ij}+F_{t,ij}\right)+m_{i}g$$

(2) $$I_{i}\frac{d\omega_{i}}{dt}=\sum_{j=1}^{k_{i}}\left(M_{t,ij}+M_{r,ij}\right)$$

where $v_{i}$ and $\omega_{i}$ are the translational and angular velocities of particle *i*, respectively. The forces considered are gravitational and particle–particle interaction forces including normal contact force $F_{n,ij}$ and tangential force $F_{t,ij}$ at the contact point.

Due to the small size of PA6 particles, the cohesive force between particles influences spreading behaviors, causing defects in the PA layer. Thus, a Hertz–Mindlin–JKR model considering the attractive force between particles is used here.

(3) $$F_{n,ij}=\left(\frac{4}{3}E^{*}\sqrt{R^{*}}\delta_{n}^{\frac{3}{2}}-4\sqrt{\pi \gamma E^{*}}a^{\frac{3}{2}}+\frac{4E^{*}}{3R^{*}}a^{3}\right)\hat{n}-2\sqrt{\frac{5}{6}}\beta \sqrt{S_{n}m^{*}}v_{ij}^{n}$$

where $\delta_{n}$ is the normal overlap; $E^{*}$ is the effective elastic modulus; $R^{*}$ is the effective radius, calculated by the radius of particle *i* and *j*; $R_{i}$ and $R_{j}$ are the Young modulus of particle *i* and *j*; $E_{i}$ and $E_{j}$ are the Poisson ratio ν of particle *i* and *j*. $S_{n}$ is the normal stiffness, and β is related to the coefficient of restitution e.

(4) $$\frac{1}{E^{*}}=\frac{\left(1-\nu_{i}^{2}\right)}{E_{i}}+\frac{\left(1-\nu_{j}^{2}\right)}{E_{j}}$$

(5) $$\frac{1}{R^{*}}=\frac{1}{R_{i}}+\frac{1}{R_{j}}$$

(6) $$S_{n}=2E^{*}\sqrt{R^{*}\delta_{n}}$$

(7) $$\beta =\frac{-lne}{\sqrt{{ln}^{2}e+\pi^{2}}}$$

where γ is the surface energy and a is the contact radius, which is related to normal overlap $\delta_{n}$, effective radius $R^{*}$, effective Young’s modulus $E^{*}$, and surface tension γ.

(8) $$\gamma =\frac{1}{2}\left(\gamma_{1}+\gamma_{2}-2\gamma_{12}\right)$$

(9) $$\delta_{n}=\frac{a^{2}}{R^{*}}-{\left(\frac{4\pi \gamma a}{E^{*}}\right)}^{1/2}$$

The equation of tangential force $F_{t,ij}$ is listed as below.

(10) $$F_{t,ij}=-\min\left(\mu_{s}F_{n},\;S_{t}\delta_{t}-2\sqrt{\frac{5}{6}}\beta \sqrt{S_{t}m^{*}}v_{t}^{\mathrm{rel}}\right)$$

where $S_{t}$ is the tangential stiffness, $v_{t}^{\mathrm{rel}}$ is the relative tangential velocity, $\mu_{s}$ is the coefficient of sliding friction, and $G^{*}$ is the shear modulus.

(11) $$S_{t}=8G^{*}\sqrt{R^{*}\delta_{n}}$$

The torque that acts on particle *i* by particle *j* includes two components. One is generated by tangential force, causing particle *i* to rotate, $M_{t,ij}$, and the other one is rolling friction torque $M_{r,ij}$ generated by normal force that acts to oppose the relative rotation between the contacting particles.

(12) $$M_{t,ij}=r_{ij}\times F_{t,ij}$$

(13) $$M_{r,ij}=-\mu_{r}\left|F_{cn,ij}\right|R_{i}\omega_{i}$$

where $r_{ij}$ is a vector pointing from the particle center and the contact point. $\mu_{r}$ is the coefficient of rolling. For a more detailed description, one can refer to Zhang et al. [23].

### 2.2. Simulation Conditions and Model Validation

**Figure 2:**
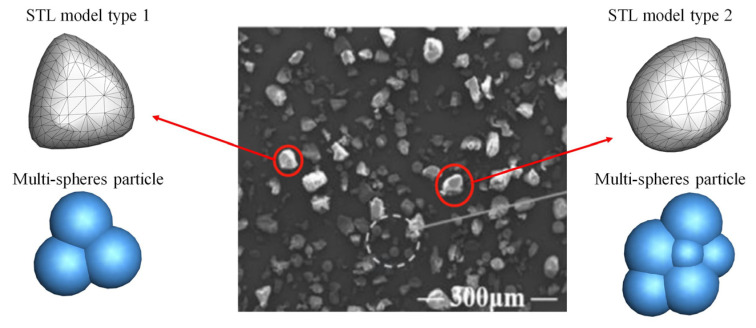
Two typical 3D-reconstructed realistic shapes of PA6 particles from SEM image.

**Figure 3:**
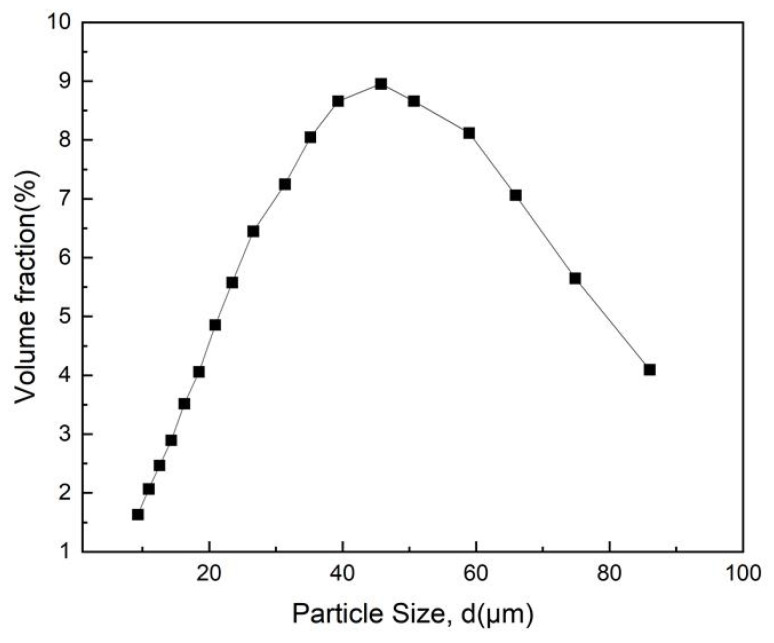
Particle size distribution of PA6 used in this work.

PA6 polymers, one of the high-performance polymers with high mechanical strength and fatigue resistance, are generated randomly in a certain area above a baseplate and are then dropped down under gravity and packed on the baseplate. A blade has a gap with the baseplate and moves under a certain velocity to spread the particles and form a powder bed. From the Scanning Electron Microscope (SEM) image of real PA6 particles (Figure 2), two typical realistic shapes are chosen and three-dimensionally reconstructed into stereo-lithography (STL) models. These STL models are filled by several spheres and form a multi-sphere particle, which can be regarded as a whole (reconstruction shape) in the DEM simulation. As shown in Figure 2, models are filled by three and six spheres. Note that, for the particle with a reconstructed shape, an equivalent diameter is calculated by the sphere diameter with the same volume. The particle size distribution is in logarithmic normal distribution, ranging from 9.86 to 86.4 μm, with an average size of 41.1 μm (d_p50_). The volume percentage and number percentage of size distribution is shown in Figure 3.

The dimension of the powder bed is L × M = 6500 μm × 1000 μm. The size of the powder bed is two orders of magnitude larger than the maximum size of the particles (~86.4 μm). The distance between the blade and baseplate is named the gap height, which is set as 4 d_p50_. To clarify the spreading behavior, a number of particles with the same size distribution in Figure 3 are produced. Then, the different spreading velocities used in our simulation are 0.02 m/s, 0.05 m/s, and 0.1 m/s in order to investigate the density of the layer. The effect of particle shape, blade velocity, layer thickness, and surface energy on powder-spreading behavior is investigated.

**Figure 4:**
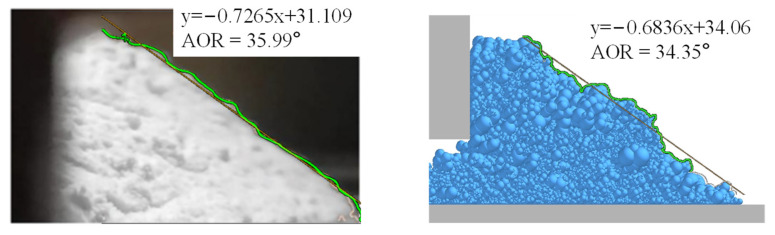
Angle of repose during spreading compared with experiment [22].

The periodic boundary is used in the y-direction. To clarify the validity and effectiveness of our simulation and reconstructed shape, a dynamic angle of repose (AOR) formed by special reconstructed particle shapes (Figure 4) is compared with the experimental result of Si et al. [22]. The angle of repose formed by reconstructed shapes is around 34.35°, which is smaller than the value of 35.99° in the experiment. Except for particle shape, the surface curves of the powder pile and AOR are in good agreement with the simulation from Si et al. [22], indicating the reconstructed shape is reliable for non-spherical particles. This result demonstrates that particle shape has a certain effect on the packing and flowing properties in the simulation. The specific simulation parameters used here are shown in Table 1.

## 3. Results and Discussion

### 3.1. Effect of Particle Shape on Bed Quality

**Figure 5:**
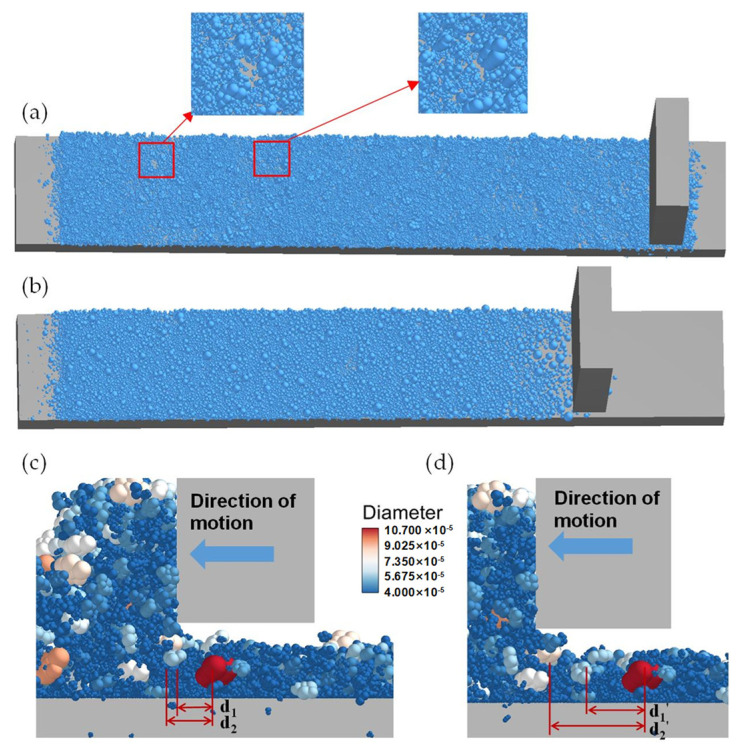
Different packing pattern of (**a**) reconstructed shape; (**b**) spherical particle. The formation of arch: (**c**) t = 0.069 s; (**d**) t = 0.072 s.

Particles with reconstructed shapes and spherical particles with the same size distribution are all spread by a blade at a certain height (1.65 mm). It should be pointed out that the spreading bed length is 6500 μm. To avoid the effect of the initial movement of the blade and the end area, the bed used for analysis is from 0.75 mm to 5.75 mm. Different from continuous material, the force distribution exists in a discontinuous variation due to the discreteness of the particle system. The powder-layer packing patterns of different particle shapes are shown in Figure 5. It can be seen that with particles with reconstructed shapes, the spreading length is longer than that of spherical particles, correspondingly illustrating low packing density. The reason for this is that some voids formed in the powder layer are composed of particles with reconstructed shapes. From the enlarged picture of the void, it can be found that after a cave, large particles with reconstructed shapes follow. This phenomenon is called an “arch”, which is also observed in metal-based particles. The formation of the arch is shown in Figure 5c,d; the color represents the diameter of the particle, in which red and pink are large-size particles, and blue represents small-size particles. The red particle is labeled 1, the light blue particle is labeled 2, the distance between 1 and 2 is d_1_, the pink particle is labeled 3, and the distance between 1 and 3 is d_2_. After a certain period, from t = 0.069 to 0.072, the pink and light blue particles have a large distance, d_1_‘ and d_2_‘ respectively. During the process in which the blade drags the large pink particles, a cave will be formed near the pink particles. In this way, large particles with reconstructed shapes are blocked below the blade, forming an arch between the blade and the bottom plate. This is because the pink particles are larger in size, and the non-spherical shaped particles cause higher rotational inertia, which are hard to roll and liable to be carried away by the blade. This is the main reason for the difference in packing pattern and porosity between spherical particles and non-spherical particles. It can be concluded that the simulation of spherical particles cannot replace non-spherical particles. In addition, the drastic fluctuation of the force arch effect leads to an uneven powder-layer structure and reduces the packing density. Thus, during the powder-spreading process, a weaker force arch is expected.

**Figure 6:**
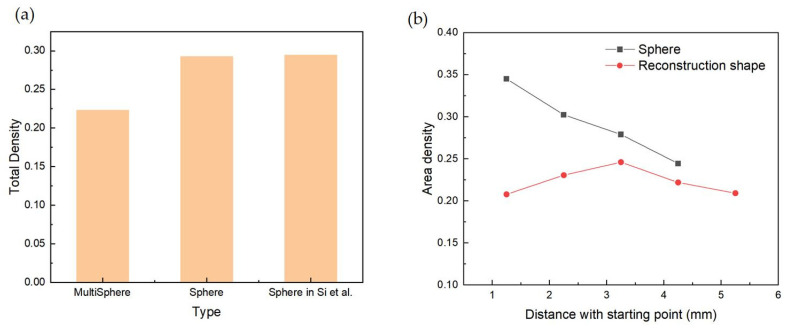
(**a**) Total density including multisphere, sphere, and sphere results from Si [22] and (**b**) density distribution at different positions along the spreading direction of spherical particles and particles with reconstructed shapes.

Higher powder-layer density results in higher laser energy absorption and denser AM parts, meaning that the powder-layer density is an important index to evaluate the powder-bed quality. The total density is calculated to analyze the distribution of particles with reconstructed shapes in Figure 6a, including cases with spheres and the spheres in Si et al.’s study. It can be found that the spherical particles have a similar density to the results achieved by Si et al. [22], which is higher than that of particles with reconstructed shapes. The area density at different positions along the spreading direction is recorded separately for spherical particles and particles with reconstructed shapes. The results of density distribution are illustrated in Figure 6b. The density of spherical particles shows a decreasing trend from the beginning area to the end area. In contrast, the density of particles with reconstructed shapes increases at first and then decreases slowly until the end area, and it reaches its peak in the central area. This result is affected by the distribution of large-size particles and the arch formed near the blade, which is in good agreement with the packing pattern (Figure 5). Since the largest diameter of the reconstructed shape is bigger than the equivalent diameter of the sphere, the arch near the blade is more significant for the reconstructed shape, which can be found in Figure 5.

**Figure 7:**
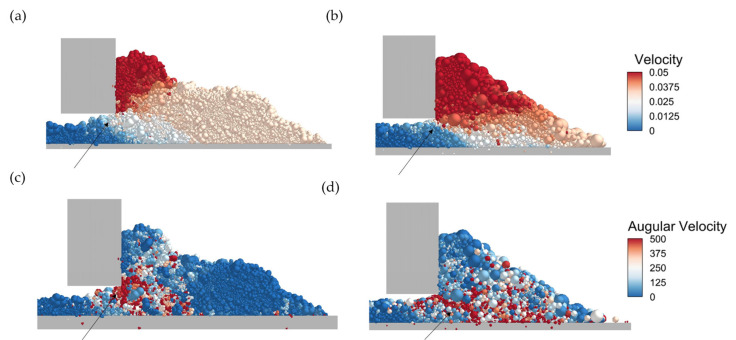
Velocity distribution of (**a**) particle with reconstructed shape and (**b**) spherical particle (The area pointed by the arrow is the particles with a velocity of around 0.025 m/s). Angular velocity distribution of (**c**) particle with reconstructed shape and (**d**) spherical particle (The area pointed by the arrow is the particles with the largest angular velocity).

The velocity and angular velocity of the particles are crucial to spreading behavior and further processing. In this regard, Figure 7a,b demonstrate that the velocity of the particles with reconstructed shapes is lower than that of spherical particles gathering in front of the blade. Interestingly, it can be found that there is a conspicuous turning point between particles with reconstructed shapes from 0.05 m/s (red) to 0.02 m/s (light red). This turning line is “right-upwards” for particles with reconstructed shapes, while it is “right-downwards” for spherical particles. Though reconstruction particles have lower velocities along the spreading direction, the velocity of reconstructed particles beneath the blade is larger than that of spherical particles, leading to large porosity. A similar phenomenon is observed in the angular velocity distribution (Figure 7c,d). The maximum angular velocity only appears near the blade for particles with reconstructed shapes, shaped “right-upwards” (red color particles). But it is a slice for spheres, pointing to the baseplate downwards, shaped “right-downwards”. This difference might be caused by the different inertia of particles with reconstructed shapes.

### 3.2. Effect of Blade Velocity

**Figure 8:**
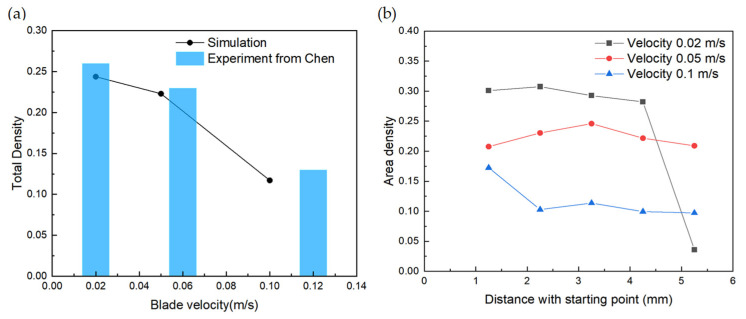
(**a**) Total density (our simulation results and experimental results from Chen [24]) and (**b**) density distribution at different positions along the spreading direction of different blade velocities.

Blade velocity is an important factor affecting the packing pattern and can be controlled easily in the experiment. Therefore, the effect of blade velocity with the sphere with a reconstructed shape is investigated here. Different blade velocities including 0.02 m/s, 0.05 m/s, and 0.1 m/s are discussed separately. The total density of different blade velocities is shown in Figure 8a, in which with the blade velocity increasing, the total density decreases. The particles velocities passing through the gap increase with the blade velocity, and the number of particles deposited on the substrate decreases, which causes the powder-layer density to deteriorate.

The detailed trend can be seen in Figure 8b, which is the density distribution along the spreading direction. For low velocity at 0.02 m/s, the density is around 0.3. When the distance to the starting point is 5 mm, there is only a small number of particles left, leading to low density in this area. For high velocity at 0.1 m/s, the density is higher at the beginning area with the distance of 1.25 mm compared with other positions. This might be caused by the initial particle-generating setting. Except for the beginning area of high velocity and the ending area of low velocity, the density is quite steady, meaning that each blade velocity leads to a corresponding density. Thus, more attention should be paid to the beginning and ending areas during each spreading process.

To investigate the effectiveness of the simulation results, the calculated variation trend is compared with the experiments conducted by Chen et al. [24]. During the experiments, typical scraping-type powder spreading is applied. It can be seen that the paved powder bed gradually decreases from 0.24 to 0.11 as the blade velocity increases from 0.02 m/s to 0.1 m/s. Moreover, Chen reported that paved powder bed becomes thinner and looser as the spreading speed increases, which is intuitive and agrees with our results.

**Figure 9:**
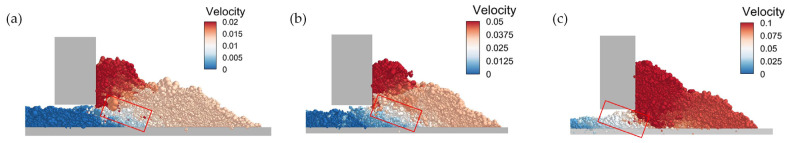
Velocity distribution of particles at different blade velocities (The velocity of the white particles in the red square is the half of the blade velocity): (**a**) 0.02 m/s, (**b**) 0.05 m/s, (**c**) and 0.1 m/s.

The velocity of powder particles during powder paving has an important influence on the final packaging quality, and the more detailed velocity distribution of particles is illustrated in Figure 9. The red color corresponds to the highest velocity, 0.02 m/s, 0.05 m/s, and 0.1 m/s. The white color marked in a red rectangle is half the highest velocity. It can be seen that the white-colored part is thin when the blade velocity is 0.02 m/s. Then, this part moves backwards until it is beneath the blade when the blade velocity is 0.1 m/s. For particles near the blade, the particles’ velocity reaches the same velocity as the blade. But for the particles at the front of the baseplate, the relative velocity increases with the increasing blade velocity, shown in light red at a low blade velocity and dark red at a high blade velocity. We can see that when the blade velocity is 0.1 m/s, particles have relatively high velocity, leading to a longer time and further distance to the end, which is the main reason for the low packing density shown in Figure 8.

### 3.3. Effect of Layer Thickness

**Figure 10:**
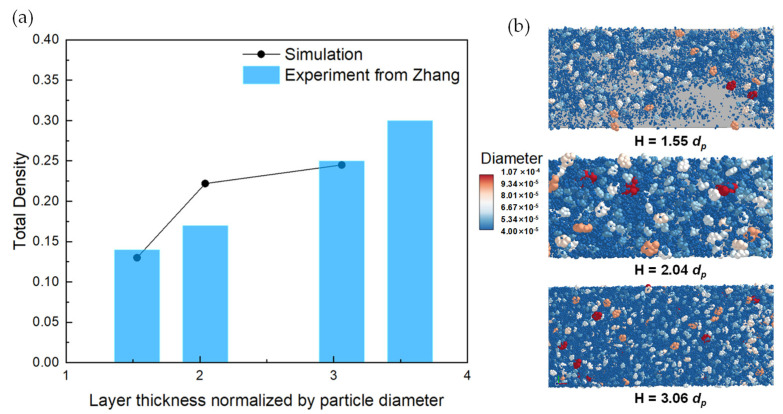
(**a**) The variation in density with layer thickness (blade height × *d_p_*) including simulation and experimental results from Zhang, (**b**) packing patterns of powder bed under different powder-layer thicknesses (1.55 *d_p_*, 2.04 *d_p_*, 3.06 *d_p_*).

Because the particle size fluctuates from 9.86 to 86.4 μm, the actual layer thickness is usually less than the solidified layer during the AM process. In this section, the influence of layer thickness on the total packing density and surface uniformity of particles with reconstructed shapes is explored, which is directly related to the spreading behavior of particles. The variation trend is plotted in Figure 10, in which the layer thickness (*H*) is set as 1.55 *d_p_*, 2.04 *d_p_*, and 3.06 *d_p_*. High values of particle diameter to layer thickness (*d_p_*/*H*) would lead to the blocking of particles, inducing discontinuous flow and more voids. As shown in Figure 10b, when H = 1.55 *d_p_*, the powder layer has many voids with uneven surfaces. With the increase in the layer thickness, the number of voids decreases gradually, and a dense powder layer with a uniform surface is obtained. Figure 10b shows the blocking behavior and cavity formation of particles during the spreading process (H = 3.06 *d_p_*), indicating that small-diameter particles are blocked by large-diameter particles in the gap near the blade. In addition, Zhang et al. precisely set the powder-layer thickness through feeler and server motors, which is the most important step in experiments [6]. Every experiment has been repeated five times to calculate average powder-layer density in order to lessen the influence of the fluctuated particle diameter. The normalized powder-layer thickness from the experiment results is divided by D50, as shown in Figure 10a. The same variation law between the numerical and experimental results indicates the reliability of the DEM model. Therefore, the actual layer thickness should be reasonably selected according to the particle size to weaken the blocking effect of large particles, so as to achieve a uniform and dense powder layer.

### 3.4. Effect of Surface Energy

**Figure 11:**
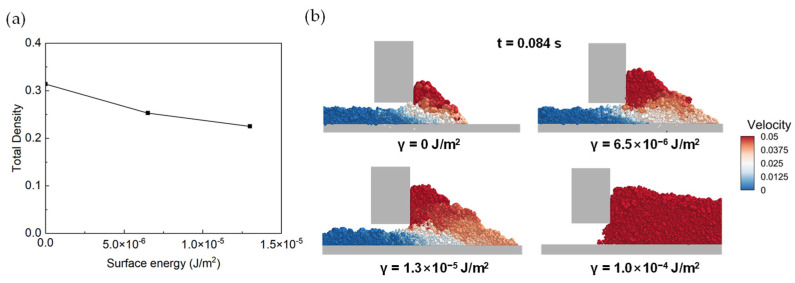
(**a**) The variation in density with surface energy, (**b**) velocity distribution of particles in the gap area under different surface energy (γ = 0, 6.5 × 10^−6^, 1.3 × 10^−5^, 1.0 × 10^−4^, respectively).

The cohesion force effect of micron dry PA6 powder on the flow behavior of powder in the spreading process cannot be ignored. Therefore, the simulated surface energy is set as 0, 6.5 × 10^−6^, 1.3 × 10^−5^, and 1.0 × 10^−4^ to investigate the density and velocity distribution. Figure 11a shows that with the increase in surface energy, the packing density decreases, which demonstrates the higher of cohesion force effect of the particles with reconstructed shapes and the poor spreading quality. A similar phenomenon is recorded in Figure 11b. When γ = 0 J/m^2^, the velocity distribution is relatively uniform and the powder layer is dense. When the surface energy is equal or larger than 6.5 × 10^−6^ J/m^2^, the decrease in powder fluidity is caused by cohesion induced by fine-particle aggregation and cavity formation, resulting in a decrease in the packing density and structural uniformity of the powder layer. Though the trend of the velocity distribution is similar, the number of particles blocked in the gap area grows with the surface energy. This means that it is more difficult for particles to spread, and the powder-layer speed is slow. This means that the density of the powder spread is low. When the surface energy increases to 1.0 × 10^−4^ J/m^2^, the particle cohesion effect is too high, resulting in an inability to spread. The simulation result suggests that a minimized particle cohesion effect is beneficial to obtain a uniform and dense powder layer. In order to optimize the PA6 non-spherical particles used, the powder-layer quality can be improved by mechanical grinding and surface modification to reduce the surface energy of particles.

## 4. Conclusions

The spreading behavior of non-spherical PA6 particles is investigated using particles with reconstructed shapes (by building multi-sphere particles) in DEM simulation, in which the effect of particle shape and blade velocity have been discussed. After blade spreading, the packing pattern reveals that large particles with reconstructed shapes are liable to form cavities and arches, which is the reason for the lower packing density compared to that of spherical particles. The variation in density with the spreading process is different: for particles with reconstructed shapes, the maximum density appears in the central area; comparatively, for spherical particles, the density keeps a downtrend with the spreading distances. A more detailed mechanism could be found from the velocity and angular velocity distribution. The shape of maximum angular velocity is “right-downwards” for particles with reconstructed shapes and “right-upwards” for spheres near the blade. The packing density decreases with the increase in blade velocity, which is caused by a longer time and further distance causing high blade velocity. The increased blade velocity makes the quality of the powder layer and subsequent parts suffer. The density is quite steady along the spreading direction, showing a uniform quantity at a certain blade velocity. Moreover, a small particle-diameter-to-layer-thickness ratio and cohesion force effect would cause increased density, which should be carefully considered during the AM process. Non-spherical and irregular particles are widely used and play an important role in processing quality. Further model development can provide an understanding of the flow behavior, enhancing the surface quality of the devices adopting powder bed-based additive manufacturing.

## Figures and Tables

**Table 1:** Material properties and parameters used in DEM simulation.

Parameters	Value
Particle shape	Reconstruction shape
Particle number, N	51,390
Particle diameter, *d_p_*	9.86–86.4 μm
Particle density, ρ_p_	1300 kg/m^3^
Young’s modulus, E	1 × 10^6^ Pa
Poisson ratio, ν	0.35
Sliding friction coefficient, μ	0.49
Rolling friction coefficient, μ_r_	0.13
Restitution coefficient, e	0.5
Time step, Δt	9 × 10^−8^ s
Surface energy, *γ*	1.3 × 10^−5^ J/m^2^

## Data Availability

The data are publicly available due to all data being contained within the article.
